# Association of Psychotherapy with Disability Benefit Claim Closure among Patients Disabled Due to Depression

**DOI:** 10.1371/journal.pone.0067162

**Published:** 2013-06-28

**Authors:** Shanil Ebrahim, Gordon H. Guyatt, Stephen D. Walter, Diane Heels-Ansdell, Marg Bellman, Steven E. Hanna, Irene Patelis-Siotis, Jason W. Busse

**Affiliations:** 1 Department of Clinical Epidemiology and Biostatistics, McMaster University, Hamilton, Ontario Canada; 2 Department of Anesthesia, McMaster University, Hamilton, Ontario, Canada; 3 Department of Medicine, McMaster University, Hamilton, Ontario, Canada; 4 National Disability Services, Policy & Procedure Department, Sun Life Financial, Toronto, Ontario, Canada; 5 Psychiatry & Behavioural Neurosciences, McMaster University, Hamilton, Ontario, Canada; Catholic University of Sacred Heart of Rome, Italy

## Abstract

**Background:**

Depression is the most frequent reason for receiving disability benefits in North America, and treatment with psychotherapy is often funded by private insurers. No studies have explored the association between the provision of psychotherapy for depression and time to claim closure.

**Methods:**

Using administrative data from a Canadian disability insurer, we evaluated the association between the provision of psychotherapy and short-term disability (STD) and long-term disability (LTD) claim closure by performing Cox proportional hazards regression.

**Results:**

We analyzed 10,508 STD and 10,338 LTD claims for depression. In our adjusted analyses, receipt of psychotherapy was associated with longer time to STD closure (HR [99% CI] = 0.81 [0.68 to 0.97]) and faster LTD claim closure (1.42 [1.33 to 1.52]). In both STD and LTD, older age (0.90 [0.88 to 0.92] and 0.83 [0.80 to 0.85]), per decade), a primary diagnosis of recurrent depression versus non-recurrent major depression (0.78 [0.69 to 0.87] and 0.80 [0.72 to 0.89]), a psychological secondary diagnosis (0.90 [0.84 to 0.97] and 0.66 [0.61 to 0.71]), or a non-psychological secondary diagnosis (0.81 [0.73 to 0.90] and 0.77 [0.71 to 0.83]) versus no secondary diagnosis, and an administrative services only policy ([0.94 [0.88 to 1.00] and 0.87 [0.75 to 0.996]) or refund policy (0.86 [0.80 to 0.92] and 0.73 [0.68 to 0.78]) compared to non-refund policy claims were independently associated with longer time to STD claim closure.

**Conclusions:**

We found, paradoxically, that receipt of psychotherapy was independently associated with longer time to STD claim closure and faster LTD claim closure in patients with depression. We also found multiple factors that were predictive of time to both STD and LTD claim closure. Our study has limitations, and well-designed prospective studies are needed to establish the effect of psychotherapy on disabling depression.

## Introduction

Major Depressive Disorder (henceforth referred to as depression) results in immense human suffering and is associated with considerable socioeconomic costs. Depression accounts for 11% of disability worldwide and an estimated productivity loss of $14.4 billion annually in Canada [1], [2], [3], [4]. The World Health Organization (WHO) estimates suggest that depression will become the second leading cause of disease burden worldwide by the year 2020 [5], [6], [7].

The National Institute for Health and Clinical Excellence (NICE) in the UK has recommended that health care professionals provide, alone or in combination, pharmacological treatments and high-intensity psychological interventions for individuals suffering from moderate or severe depression. The most frequently prescribed psychotherapy for treating depression is cognitive behavioural therapy (CBT) [8], [9].

Depression is a common reason for receiving disability benefits [10], [11], [12], incurring more costs for long-term disability (LTD) than other disorders [13]. Individuals suffering from psychiatric disorders who are also receiving disability benefits require more complex treatment and have more difficulty returning to work than those suffering from other disabling complaints [14]. Psychological therapy may be less effective, or ineffective, in patients receiving disability benefits, as their circumstances or psychological status may interfere with the successful implementation of therapy [15]. There is indirect evidence for this hypothesis from surgical populations: a recent meta-analysis of 129 studies revealed that the odds of an unsatisfactory outcome in patients receiving disability benefits or engaged in litigation was 3.79 times greater (95% confidence interval [CI]: 3.28 to 4.37) versus similar patients not in receipt of disability benefits or pursuing litigation [16].

Given that psychotherapy is one of the most frequently reimbursed treatment for depression by private insurers [17], it is important to ascertain if psychotherapy represents a worthwhile expenditure of time and energy for depressed patients, and a good investment for insurers. We recently completed a systematic review in which none of 92 randomized controlled trials (RCTs) that explored the effect of CBT on depression reported whether enrolled patients were receiving disability benefits. We successfully contacted 56 trialists and identified 3 trials that captured information on disability benefit status [18]. Our analyses consisting of 2 trials (including 34 patients on disability benefits) did not find a significant difference in depression between patients receiving disability benefits versus those not receiving disability benefits. However, we were limited by the small number of patients available for analysis.

In the present study, we used the administrative data of a large Canadian, private, disability insurer (Sun Life Financial Inc.) to explore the association between the provision of psychotherapy for patients suffering from depression and time to both short-term disability (STD) and LTD claim closure. Additionally, we evaluated what factors were associated with receipt of psychotherapy in patients with depression in receipt of disability benefits.

## Methods

### Ethics statement

The Research Ethics Board at McMaster University approved our study. The ethics board waived the need for written informed consent, as this was retrospective study where the de-identified data were analyzed anonymously.

### Design

Secondary analysis of an insurance administrative database

### Description of patients and eligibility criteria

Between January 2007 and December 2010, Sun Life Financial had 259,510 claims submitted for approval. Of these, 190,527 were STD claims and 68,983 LTD claims. An STD and LTD claim differ in regards to the potential duration of time that claimants may receive wage replacement benefits. The two most common standard benefit periods for an STD claim is up to 17 weeks and 26 weeks, but some plan benefit periods may be less or more than this duration. An LTD claim pay wage replacement benefits for a longer period, up to age 65.

Of the 259,510 filed claims at Sun Life, 172,425 (90.5%) STD and 55,530 (80.5%) LTD claims were approved. For our analyses, we included all claims (1 claim per claimant) that were approved for STD or LTD benefits with a primary diagnosis of major depressive disorder or recurrent depressive disorder. We excluded all individuals whose claims were recorded as closed prior to contractual approval, and STD claims with a maximum claim benefit period over 2.5 years as we deemed those to be data entry errors.

### Administrative Variables

The database consisted of demographic, administrative, and clinical information. The case manager(s) responsible for overseeing each claim entered all data. The standard requirement is for data to be entered within 5 days of claim receipt for STD claims, and within 10 days for LTD claims, although this may vary.

Guided by the results from observational studies evaluating predictors of recovery in patients receiving disability benefits due to depression [19], [20], [21], and a systematic review evaluating prognostic factors of long term disability due to mental disorders [22], we selected, *a priori*, 14 variables from the database that we judged may be associated with claim closure and receipt of psychotherapy, and predicted the direction of anticipated effects. In addition to the variables we chose from previous evidence, we included and predicted the direction of anticipated effects of two additional variables (time to claim approval and disability funding policy) in our model, as per recommendations by content experts in our research team and the administrative team at Sun Life. Two psychologists, blinded to study results, provided hypotheses on the anticipated direction of effect of receipt of psychotherapy on STD and LTD claim closure. They predicted that individuals in the STD group are less likely to benefit from psychotherapy given the time taken for CBT to be successfully implemented, which may take them into the LTD timeframe. They predicted that individuals in the LTD group are more likely to benefit from psychotherapy. Further, they hypothesized that if there was a difference in the anticipated directions between the two groups, it may be due to the increased severity of illness or secondary gains in those receiving psychotherapy in the STD group. Table 1 provides a description of all independent variables considered in our models and our predictions on the anticipated direction of effect on disability claim closure.

**Table 1:** Description of variables

Variable	Description	Anticipated direction of claim closure
*Claimant demographic variables*		
Age	Age of claimant at disability	Older age: (−)
Gender	Gender of claimant	Females: (−)
Province	Province the claimant resides in	Ontario, Quebec: (+)
Industry	Type of industry the claimant is working in (blue collar, grey collar, white collar*)	White, grey collar: (−)
Salary	Salary of claimant	Higher salary: (−)
ICD-10 Primary Diagnosis	Primary diagnosis of claimant (major depression or recurrent depression)	Recurrent depression: (−)
ICD-10 secondary diagnosis	Secondary diagnosis of claimant (none, psychological diagnosis, non-psychological diagnosis)	Secondary diagnosis: (−)
*Claim coverage variables*		
Time to claim registration	Duration from claimant’s disability date to disability claim registration date	Longer time to claim registration: (−)
Time to claim approval	Duration from disability claim registration date to disability claim contractual approval date	Longer time to claim approval: (−)
Elimination period	Duration from claimant’s disability date to first payment date	Longer elimination period: (−)
Maximum claim benefit period	Duration from disability claim contractual approval date to maximum claim benefit date	Longer claim benefit period: (−)
Location of claim office	Office where the claim is currently managed (Edmonton, Montreal, Toronto, Vancouver, Waterloo)	Toronto, Montreal: (+)
Funding type	Funding arrangement of claim (non-refund, refund, administrative services only [ASO])	Refund, ASO: (−)
Total reserve amount	Reserves held on claims (LTD only)	Higher reserves: (+)
Receipt of psychotherapy	If claimant has received psychotherapy or not	Receipt of psychotherapy: (O for STD/+for LTD)

ICD: International classification of diseases; +: associated with faster claim closure; −: associated with slower claim closure; O: associated with similar resolution; * − classifications of industry in Table S1; LTD – long term disability

Disability funding policies can be purchased by employers under three types of financial arrangements: non-refund policies where the insurer approves and funds services and treatments, refund policies in which the insurer and the plan sponsor (e.g., the employer) shares the funding for services and treatments, and administrative services only (ASO) policies in which the plan sponsor approves and pays for all services and treatments. For LTD claims, all types of policies require funds to be put aside (as reserves) that amount to two-thirds of the claimant's pre-disability income that would be earned from the age their long-term benefits began until the age of 65. Under a non-refund policy, the reserves are funded by the insurer, and released back to the insurer if the claim resolves. Under refund or ASO policies, the reserves for LTD claims are funded by the employer, and released back to the employer if the claim resolves. For our regression models, we used non-refund policy as the reference group.

Two authors (SE and JWB) independently grouped 66 different industries into blue-collar, grey-collar and white-collar industries (94% agreement) and reached consensus through discussion (Table S1).

### Outcomes

Our primary outcome was time to claim closure, defined as the duration from disability claim approval until the closure/resolution of the claim. Our secondary outcome was receipt of psychotherapy.

### Data management and data cleaning

We screened all data to identify outliers, inconsistencies and missing data by calculating summary statistics, and exploring distributions graphically. If clear outliers and inconsistencies were identified, we worked with Sun Life Financial to correct the data. If inconsistencies could not be corrected, we treated them as missing data. We excluded variables that were missing for more than 10% of claimants. Of the variables that were not excluded, less than 1% was missing.

### Statistical analysis

We generated frequencies for all collected data. We reported the mean and standard deviation (SD) of continuous variables that were normally distributed, the median and interquartile range (IQR) for continuous variables that were not normally distributed (assessed through residual analysis and computing kurtosis and skewness measures [skewness and kurtosis measures of +2 to -2 considered normal]), and the number of occurrences with proportions represented as percentages for categorical variables.

We tested for collinearity to assess if a predictor was highly correlated with another (correlation coefficient r>0.5) using a correlation matrix. If two variables were highly correlated, we removed the variable that was considered to be of lesser importance, as guided by the administrators at Sun Life Financial and content experts on our team.

We performed a time-to-event analysis using Cox proportional hazards regression to assess the association between time to claim closure and the independent variables. Receipt of psychotherapy was treated as a time-dependent covariate to account for when it was initiated during the course of the disability claim. For STD claims that were unresolved 26 weeks after claim approval, we used 181 days (26 weeks [the more common STD benefit duration] minus 1 day) as our censoring point to maintain the proportionality assumption. For LTD claims that were unresolved when the data was extracted, we used the date of data extraction as our censoring point. For our secondary analysis, we performed an adjusted logistic regression to assess the association between receipt of psychotherapy and potentially predictive factors. To avoid overfitting our models, we required at least 10 events per variable for our Cox regression model and 10 events of the least common outcome—receipt of psychotherapy—for our logistic regression model [23]. Our regression model excludes independent variables with less than 200 observations unless we were able to collapse them with other related variables to exceed this threshold. We calculated hazard ratios (HRs) for our time-to-event analyses and odds ratios (Ors) for our logistic regression analyses, their associated 99% confidence intervals (Cis), the unstandardized beta coefficients for each variable and the associated p-values. In order to be more stringent and minimize the likelihood of spurious findings, we considered an independent variable as statistically significant if it had a p-value of less than or equal to 0.01 in each final adjusted model.

We performed bootstrapping for our regression models to measure the accuracy of our sample estimates [24], and performed the Hosmer-Lemeshow test to assess the goodness-of-fit in our logistic regression model.

We used SPSS v20.0 to perform all statistical analyses.

## Results

Of 13,758 STD and 11,275 LTD claims received with a primary diagnosis of depression, 3250 (24%) STD and 937 (8%) LTD claims were excluded due to the claim being declined, exceeding a claim benefit period of 2.5 years (STD only), or having their claim paid retroactively. Our final analysis included 10,508 STD and 10,338 LTD claims. Depression management included psychotherapy in 261 STD claims and 1,582 LTD claims. Table 2 presents baseline characteristics of all factors for STD and LTD claims.

**Table 2:** Baseline characteristics

Variables	STD n(%)	LTD n(%)
Total claimants	10508	10338
Claim closure reason		
Censored at time of data extraction	173 (1.6%)	3670 (35.5%)
Return to work	3390 (32.3%)	4542 (43.9%)
Anticipated return to work	2900 (27.6%)	0 (0%)
Maximum benefit date reached	3003 (28.6%)	0 (0%)
Offsets exceed benefits	4 (0.04%)	8 (0.1%)
Retirement/terminal age	11 (0.1%)	141 (1.4%)
Own occupation termination	0 (0%)	451 (4.4%)
No longer disabled	305 (2.9%)	1092 (10.6%)
Claim transfer or settlement	0 (0%)	57 (0.6%)
Rehabilitation settlement	0 (0%)	280 (2.7%)
Litigation settlement	0 (0%)	31 (0.3%)
Other (including strike and securing info)	715 (6.8%)	20 (0.2%)
Death	7 (0.1%)	41 (0.4%)
Age: Mean (SD) years	43.3 (10.1)	47.0 (9.3)
Sex		
Male	5859 (55.8%)	3380 (67.3%)
Female	4640 (44.2%)	6958 (32.7%)
Salary per month: Median (IQR)	$3337 ($2607 to $4450)	$4344 ($3204 to $5154)
Industry		
White-collar	2502 (23.8%)	2904 (28.1%)
Grey-collar	3474 (33.1%)	2112 (20.4%)
Blue-collar	4532 (42.1%)	2490 (24.1%)
Unknown*	0 (0%)	2832 (27.4%)
Primary diagnosis		
Major depression	10004 (95.2%)	9034 (87.4%)
Recurrent depression	504 (4.8%)	1304 (12.6%)
Secondary diagnosis		
None	8043 (76.5%)	6657 (64.4%)
Psychological diagnosis	1746 (16.6%)	2337 (22.6%)
Nonpsychological diagnosis	719 (6.8%)	1344 (13.0%)
Province		
British Columbia	842 (8.0%)	966 (9.3%)
Alberta**	1736 (16.5%)	1112 (10.8%)
Saskatchewan**	237 (2.2%)	119 (1.2%)
Manitoba**	155 (1.5%)	155 (1.5%)
Ontario	3798 (36.1%)	3532 (34.2%)
Quebec	2956 (28.1%)	3922 (37.9%)
New Brunswick***	105 (1.0%)	189 (1.8%)
Nova Scotia***	537 (5.1%)	224 (2.2%)
Prince Edward Island***	5 (0.05%)	35 (0.3%)
NewFoundland***	139 (1.3%)	83 (0.8%)
Claim office		
Vancouver	507 (4.8%)	798 (7.7%)
Edmonton	1953 (18.6%)	959 (9.3%)
Waterloo	2702 (25.7%)	1534 (14.8%)
Toronto	1707 (16.2%)	1084 (10.5%)
Montreal	3639 (34.6%)	5963 (57.6%)
Funding type		
ASO	4042 (38.5%)	645 (6.2%)
Non-Refund	3376 (32.1%)	4399 (42.6%)
Refund	3090 (29.4%)	5294 (51.2%)
Time to claim receipt months: median (IQR)	0.3 (0.2 to 0.6)	4.0 (2.8 to 5.3)
Time to claim approval months: median (IQR)	0.4 (0.2 to 0.7)	1.5 (0.9 to 2.3)
Reserve amount: median (IQR)	N/A	$283,678 ($121,742 to $629,813)
Receipt of psychotherapy		
No	10247	8756
Yes	261	1582
Time to initiate psychotherapy weeks:median (IQR)	10.1 (6.8 to 14.9)	17.1 (8.7 to 34)

STD- Short term disability; LTD– Long-term disability; SD– Standard deviation; IQR– Interquartile range;; * - Excluded from analyses; ** - merged as ‘Prairies’ for our analyses; *** - merged as ‘Maritimes’ for our analyses; N/A – not applicable due to no reserves for STD

We excluded the following variables from our regression models: total reserve amount for STD model as there are no reserves put aside for STD claims; claim office due to high correlation with province of residence; time to receipt of disability benefits and elimination period (date of disability to the date the claim was first paid) due to high correlation with time to claim approval; and type of employment industry from our LTD models due to a high frequency (27.4%) of missing data. Due to small numbers of observations from some provinces, we merged Prince Edward Island, Newfoundland, Nova Scotia and New Brunswick into one category, “Maritimes”, and Alberta, Saskatchewan and Manitoba into “Prairies”. We excluded Yukon and Northwest Territories as they had fewer than 200 observations and merging them with other provinces was not considered appropriate.

### Short-term disability

Of 10,508 STD claims due to depression, 10,335 (98.4%) were closed prior to a maximum STD benefit duration of 26 weeks and 173 (1.6%) were censored. Of the 10,335 closed claims, 3390 (32.8%) returned to work and 2900 (28.1%) were expected to return to work (Table 2).

**Figure 1:**
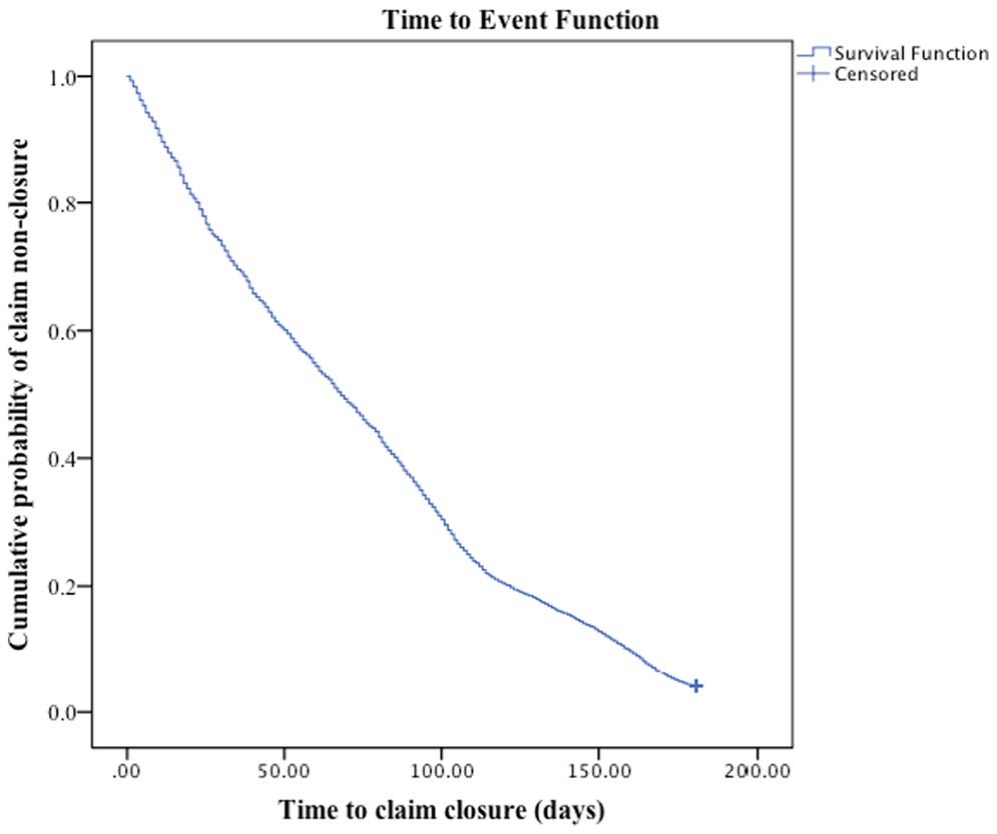
Kaplan Meier time to event curve of time to short-term disability claim closure

Figure 1 presents the time to closure survival curve for STD claimants.

#### Factors associated with time to STD claim closure

Our adjusted regression analysis showed that psychotherapy was associated with longer time to STD claim closure (HR [99% CI] = 0.81 [0.68 to 0.97]). Older age (0.90 [0.88 to 0.92], per decade), female gender (0.92 [0.87 to 0.97]), working in a white-collar industry (0.86 [0.80 to 0.92]), higher salary (0.87 [0.82 to 0.93], a primary diagnosis of recurrent depression versus non-recurrent major depression (0.78 [0.69 to 0.87], a psychological secondary diagnosis (0.90 [0.84 to 0.97] or a non-psychological secondary diagnosis (0.81 [0.73 to 0.90]), a longer maximum claim benefit period duration (0.87 [0.86 to 0.88], and an administrative services only (ASO) ([0.94 [0.88 to 1.00]) or refund policy (0.86 [0.80 to 0.92]) compared to non-refund policy claims, and residing in Quebec compared to Ontario (0.73 [0.68 to 0.78] were independently associated with slower STD claim closure (Table 3).

**Table 3:** Factors predictive of time to short-term disability claim closure

Factor	p-value	HR	99.0% CI for HR	
			Lower	Upper
Receipt of psychotherapy	.002	.813	.684	0.966
Age (per 10 years)	<.001	.902	.878	.926
Gender				
Female	<.001	.915	.865	.968
Male (reference group)		1		
Industry				
White collar	<.001	.848	.790	.911
Grey collar	.013	.941	.884	1.002
Blue collar (reference group)		1		
Salary (per $1000 per week)	<.001	.872	.815	.934
ICD-10 primary diagnosis				
Major depression (reference group)		1		
Recurrent depression	<.001	.776	.687	.876
ICD-10 secondary diagnosis				
Psychological diagnosis	<.001	.904	.842	.971
Non-Psychological diagnosis	<.001	.814	.733	.904
None (reference group)		1		
Time to approval (months)	<.001	1.105	1.065	1.147
Maximum benefit period (months)	<.001	.870	.859	.881
Province				
British Columbia	.434	1.031	.931	1.142
Prairies*	.010	1.077	1.000	1.160
Quebec	<.001	.713	.666	.764
Maritimes**	<.001	1.188	1.068	1.320
Ontario (reference group)		1		
Funding type				
ASO	.010	.939	.881	1.000
Refund	<.001	.855	.798	.915
Non-refund (reference group)		1		

HR – hazard ratio; CI –confidence interval; ICD-10 – International Classification of Diseases version 10; * - Consists of Alberta, Saskatchewan, and Manitoba; ** - Consists of New Brunswick, NewFoundland, Nova Scotia and Prince Edward Island; ASO – Administrative Services Only

Factors that have a p-value of less than 0.01 are significant predictors of claim closure

An HR of greater than 1 is associated with faster claim closure; an HR of less than 1 is associated with slower claim closure

Longer time from claim registration to claim approval (1.11 [1.07 to 1.15]), and residing in the Prairies (1.08 [1.00 to 1.16]) or the Maritimes (1.19 [1.07 to 1.32]) compared to Ontario were associated with faster STD claim closure (Table 3).

#### Factors predictive of receipt of psychotherapy among STD claims

Working in a white-collar industry (OR [99% CI] = 2.59 [1.64 to 4.08]) or a grey-collar industry (1.99 [1.27 to 3.13]) compared to a blue-collar industry, and a refund policy compared to a non-refund policy claim (1.58 [1.05 to 2.38]) were associated with a higher likelihood of receiving psychotherapy while in receipt of STD benefits.

### Long-term disability

Of 10,338 LTD claims due to depression, 6668 (65%) were closed and 3670 (35%) were censored when our data was captured. Of the 6668 closed claims, 4542 (68.1%) returned to work (Table 2).

**Figure 2:**
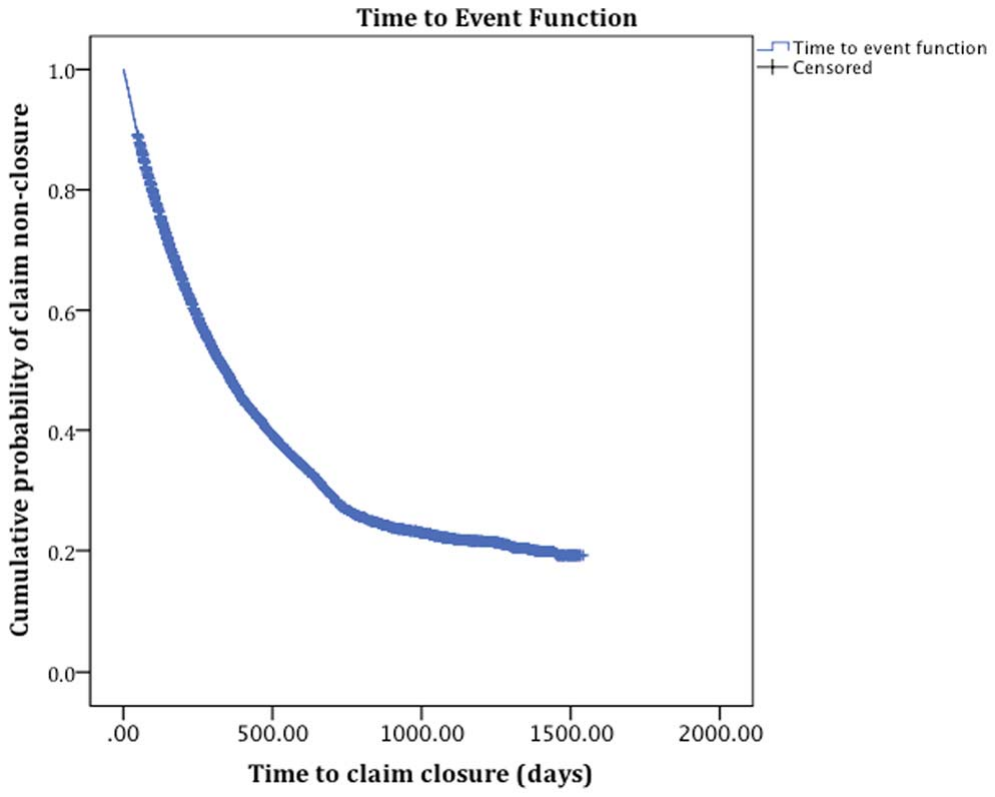
Kaplan Meier time to event curve of time to long-term disability claim closure

Figure 2 presents the time to closure survival curve for LTD claimants.

#### Factors predictive of time to claim closure

Our adjusted regression analysis showed that receipt of psychotherapy (HR [99% CI] = 1.42 [1.30 to 1.55]) was independently associated with faster claim closure. Older age (0.83 [0.80 to 0.85]), a primary diagnosis of recurrent depression (0.80 [0.72 to 0.89]), a psychological secondary diagnosis (0.66 [0.61 to 0.71]) or non-psychological secondary diagnosis (0.77 [0.71 to 0.83]), longer time from claim registration to claim approval (0.98 [0.96 to 0.997]), and ASO (0.87 [0.75 to 0.996]) or refund (0.73 [0.68 to 0.78]) policy claims compared to non-refund policy claims were associated with slower claim closure.

Residing in the Prairies (1.46 [1.31 to 1.61]) or Quebec (1.93 [1.78 to 2.09] versus Ontario were associated with faster claim closure (Table 4).

**Table 4:** Factors predictive of time to long-term disability claim closure

	p-value	HR	99.0% CI for HR	
			Lower	Upper
Receipt of psychotherapy	<.001	1.417	1.297	1.547
Age (per 10 years)	<.001	.825	.797	.854
Gender				
Female	.091	.956	.892	1.024
Male (reference group)		1		
Salary (per $1000 per week)	.278	1.076	.904	1.280
ICD-10 primary diagnosis				
Major depression (reference group)		1		
Recurrent depression	<.001	.803	.724	.891
ICD-10 secondary diagnosis				
Psychological diagnosis	<.001	.765	.705	.830
Non-psychological diagnosis	<.001	.659	.594	.732
None (reference group)		1		
Time to approval (months)	.003	.978	.959	.997
Province				
British Columbia	.197	.937	.823	1.067
Prairies*	<.001	1.455	1.313	1.611
Quebec	<.001	1.928	1.782	2.086
Maritimes**	.613	.968	.820	1.143
Ontario (reference group)		1		
Funding type				
ASO	.008	.866	.754	.996
Refund	<.001	.729	.681	.780
Non-refund (reference group)		1		

HR – hazard ratio; CI –confidence interval; ICD-10 – International Classification of Diseases version 10; * - Consists of Alberta, Saskatchewan, and Manitoba; ** - Consists of New Brunswick, NewFoundland, Nova Scotia and Prince Edward Island; ASO – Administrative Services Only

Factors that have a p-value of less than 0.01 are significant predictors of claim closure

An HR of greater than 1 is associated with faster claim closure; an HR of less than 1 is associated with slower claim closure

#### Factors predictive of receipt of psychotherapy among LTD claims

Older age (OR [99% CI] = 0.90 [0.83 to 0.97]), a non-psychological secondary diagnosis (0.78 [0.62 to 0.98]), residing in Quebec compared to Ontario (0.53 [0.44 to 0.64]), and an ASO (0.58 [0.42 to 0.80]) or refund (0.70 [0.60 to 0.82]) policy claims compared to non-refund were associated with a lower likelihood of receiving psychotherapy. Females versus males (1.20 [1.02 to 1.41]), and residing in the prairies versus Ontario (1.32 [1.08 to 1.62]) were associated with a higher likelihood of receiving psychotherapy.

Table 5 provides a summary of factors that were independently associated with time to STD and LTD claim closure compared to our anticipated direction of effect.

**Table 5:** Comparison between predictors associated with time to claim closure for short-term disability versus long-term disability claims

Predictors associated with time to claim closure	STD	LTD	Anticipated direction
**Receipt of psychotherapy**	**−**	+	O/+
Older age	−	−	−
Female (compared to males)	−	NS*	−
White collar industry (compared to blue collar industry)	−	N/A	−
Grey collar industry (compared to blue collar industry)	NS*	N/A	−
Higher salary	−	NS**	−
Recurrent depression (compared to major depression)	−	−	−
Secondary psychological diagnosis (compared to no diagnosis)	−	−	−
Secondary nonpsychological diagnosis (compared to no diagnosis)	−	−	−
Longer maximum benefit period duration	−	N/A	−
**Longer time from claim registration to claim approval**	**+**	−	−
**Prairies (compared to Ontario)**	**+**	**+**	−
**Quebec (compared to Ontario)**	**−**	**+**	O
**Maritimes (compared to Ontario)**	**+**	NS*	−
ASO funding (compared to non-refund)	−	−	−
Refund funding (compared to non-refund)	−	−	−

Bold rows represent associations that were in the opposite directions of what we anticipated

STD: short-term disability; LTD: long-term disability; +: associated with faster claim closure; -: associated with slower claim closure; O: associated with similar resolution; NS*: not significant but consistent with anticipated direction; NS**: not significant and not consistent with anticipated direction; N/A =  not included in the model

## Discussion

### Summary of main results

Our study, evaluating the effect of psychotherapy on disability benefit claim closure in patients suffering from a primary diagnosis of depression, found that receipt of psychotherapy was associated with longer time to STD closure and faster LTD claim closure. For STD and LTD claims, older age, a primary diagnosis of recurrent depression (compared to major depression), a secondary psychological or non-psychological diagnosis (versus no secondary diagnosis), and an administrative services only or a refund policy (compared to a non-refund policy) were commonly predictive of slower claim closure. We found no common predictors that were independently associated with receipt of psychotherapy for both STD and LTD claims.

### Strengths and limitations

The strengths of our study included *a priori* creation of regression models and the anticipated direction of included independent variables. Other strengths include limited missing data, correction of identifiable data errors and inconsistencies, and validation checks to ensure the accuracy of our sample estimates from our regression models.

Our study has several limitations. First, this was a retrospective cohort study in which the reasons for administering psychotherapy are uncertain. Thus, despite our adjusted models, it remains possible that selection bias affected our findings; STD claimants who received psychotherapy were less likely to resolve their claims and LTD claimants chosen to receive psychotherapy were more likely to resolve their claims irrespective of the intervention. Second, a number of variables that may be important to consider were unavailable (e.g., baseline severity of depression, patient expectations regarding recovery, and the use of antidepressants), and some variables were not optimally collected. For example, all psychotherapies were categorized as an aggregate variable and the specific type of psychotherapy provided was not available, although the insurer felt that majority of psychotherapy administered was CBT. Third, our association between psychotherapy and longer time to STD claim closure may represent a misleading finding: the effects of some common forms of psychotherapy (e.g., CBT) typically take months [i.e., greater than 3 months] to manifest [25], meaning that even an effective therapy may not show an effect during the limited time that an STD claim is paid out. Further, it is possible that those receiving psychotherapy in the STD group had more severe depression or may have been motivated by secondary financial gains (e.g., being approved for LTD benefits). Finally, our primary outcome, claim closure, reflects to only a limited extent the more important outcome of sustained return to work [26].

### Findings in context with previous evidence

The association between receipt of psychotherapy and claim closure or return to work has received limited attention in the published literature. However, a recent article reported the effectiveness of a pilot vocationally oriented CBT in assisting very long-term unemployed individuals return to work [27], and a recent RCT reported that individuals treated with work-focused CBT returned to work on average 65 days earlier than those receiving traditional CBT [28].

Our findings are consistent with previous evidence that shows that older age [22], [29], [30], [31], higher salary [29], and comorbidities (presence of secondary diagnoses) are associated with worse recovery [31], [32]. We found inconsistent association of female gender with claim closure between STD and LTD claims. Previous evidence is also inconsistent with a recent review finding no significant association [22] and two other reviews finding female gender as a significant predictor of longer duration of sick leave [29], [33]. Differences in the results may be explained by the different conditions studied and/or adjusting for different prognostic factors in the regression models.

We found that longer claim approval time was associated with longer time to LTD claim closure but faster STD claim closure, and ASO or refund policies were associated with longer time to claim closure in both STD and LTD claims. These associations had not been previously reported. Longer approval times for LTD claims may delay treatment initiation, which potentially delays recovery (and claim closure). Although we found an opposite association for STD claims, STD approval times were found to be relatively short (median (interquartile range [IQR]) of 0.4 [0.2 to 0.7] months) compared to LTD approval times (1.5 [0.9 to 2.3] months), and thus this may not represent an important effect.

The association of ASO or refund policies with delayed recovery may be explained by differences in the amount of rehabilitation services provided. For example, among LTD claims, we found that non-refund policies were significantly more likely to receive psychotherapy than other policy types: 13% of claimants with ASO and refund policies received psychotherapy compared to 18% of claimants with a non-refund policy. In our model, we were only able to adjust for psychotherapy and there could be differences in other services (e.g., work hardening program) provided between the policy types. These findings warrant further exploration.

Although we did not predict that the Prairies region would have faster claim closure in both STD and LTD claims and Quebec would have faster claim closure in LTD claims (compared to Ontario), post-hoc discussions with insurance administrators suggested that these associations may be explained by the economic growth in the Prairies region. These discussions also suggested that greater number of mental health claims treated in Quebec may have resulted in a more established infrastructure facilitating claim resolution. However, we are uncertain as to why the same effect of faster claim closure in Quebec was not illustrated for STD claims.

### Future directions

Our findings reveal uncertainty about the true effect of psychotherapy on time to claim resolution, among patients with depression. A prospective study with careful measurement of all putative determinants of claim resolution would strengthen the evidence; a randomized controlled trial could settle the issue definitively. Such research is required before payers and clinicians can confidently decide whether they should decrease, continue or expand the use of psychotherapy in managing these patients.

## Conclusions

Our study found, paradoxically, that receipt of psychotherapy is significantly associated with longer time to claim closure in individuals receiving STD, and faster claim closure in patients receiving LTD. We also found evidence to suggest that age, presence and type of diagnoses, type of policy funding, gender, salary, industry, time to claim approval and province claimants reside in are predictive of claim closure. Establishing the causal effect of psychotherapy on claim resolution will require well-designed prospective studies.

## Supporting Information

Table S1 Classification of industry
(DOC)
