# Correction: Association of Psychotherapy with Disability Benefit Claim Closure among Patients Disabled Due to Depression

**DOI:** 10.1371/annotation/fd8ade2b-e9ed-4f75-8e32-5b327535306c

**Published:** 2014-01-03

**Authors:** Shanil Ebrahim, Gordon H. Guyatt, Stephen D. Walter, Diane Heels-Ansdell, Marg Bellman, Steven E. Hanna, Irene Patelis-Siotis, Jason W. Busse

There was an error in the last sentence of the Results section of the Abstract. The correct sentence is: In both STD and LTD, older age (0.90 [0.88 to 0.92] and 0.83 [0.80 to 0.85]), per decade), a primary diagnosis of recurrent depression versus non-recurrent major depression (0.78 [0.69 to 0.87] and 0.80 [0.72 to 0.89]), a psychological secondary diagnosis (0.90 [0.84 to 0.97] and 0.66 [0.61 to 0.71]), or a non-psychological secondary diagnosis (0.81 [0.73 to 0.90] and 0.77 [0.71 to 0.83]) versus no secondary diagnosis, and an administrative services only policy ([0.94 [0.88 to 1.00] and 0.87 [0.75 to 0.996]) or refund policy (0.86 [0.80 to 0.92] and 0.73 [0.68 to 0.78]) compared to non-refund policy claims were independently associated with longer time to claim closure. 

