# Supplementary material for: Association of Psychotherapy with Disability Benefit Claim Closure among Patients Disabled Due to Depression
Source: PLoS One. 2013 Jun 28;8(6):e67162. doi: 10.1371/journal.pone.0067162 (PMC3696037; doi:10.1371/journal.pone.0067162)
Supplement: Table S1 — Classification of industry (DOC) [file pone.0067162.s001.doc]

**Table S1. Classification of industry**

| **White collar** | **Grey collar** | **Blue collar** |
| --- | --- | --- |
| Banking  Business services  Credit agencies  Educational service  Federal government  Holding related investments  Insurance agent, broker, service  Insurance carriers  Legal services  Local government  Medical, related health  Nonprofit membership organization  Provincial government  Real estate  Social services | Apparel & other finished products of fabrics & similar material  Communication  Eating drinking places  Food stores  Furniture, home furnishings  General merchandise  Hotels, rooming houses, camps & other lodging places  Local passenger train  Miscellaneous retail stores  Miscellaneous services  Motion pictures  Personal services  Printing, publishing  Retail-apparel & accessory stores  Security & commodity brokers, dealers, exchanges & services  Services-amusement & recreation services  Transportation by air  Transportation service  Water transportation  Wholesale durables  Wholesale nondurables | Agricultural production-livestock & animal specialties  Auto dealers & gasoline stations  Automotive repair, services & parking  Bituminous coal & lignite mining  Building material & farming equipment  Chemicals, allied products  Electrical equipment  Electricity, gas, sanitary service  Fabricated metal products  Food and kindred products  Forestry  General building Construction  Heavy construction  Instruments and related products  Lumber, wood products  Metal mining  Mining & quarrying of nonmetallic minerals (no fuels)  Miscellaneous manufacturing  Miscellaneous repair service  Oil gas extraction  Paper allied products  Primary metals  Railroad transportation  Rubber & plastics  Special trade contractors  Stone Clay, glass products  Textile mill products  Tobacco manufacturers  Transportation equipment  Trucking, warehousing |
